# A TRPC3 blocker, Pyr3, prevents stent-induced arterial remodeling

**DOI:** 10.1186/2050-6511-13-S1-A85

**Published:** 2012-09-17

**Authors:** Sarah König, Sara Browne, Heinrich Mächler, Gerald Höfler, C Oliver Kappe, Toma N Glasnov, Marlen Braune, Eric Wittchow, Klaus Groschner

**Affiliations:** 1 Institute of Biophysics, Medical University of Graz, 8010 Graz, Austria; 2 Department of Cardiac Surgery, Medical University of Graz, 8036 Graz, Austria; 3 Institute of Chemistry, Karl-Franzens University of Graz, 8010 Graz, Austria; 4 Institute of Pathology, Medical University of Graz, 8036 Graz, Austria; 5 Vascular Interventions R&D Group, BIOTRONIK SE & Co. KG, 91052 Erlangen, Germany

## Background

TRPC-mediated Ca^2+^ entry has been implicated in the control of smooth muscle proliferation and might represent a pivotal mechanism underlying in-stent restenosis. As we have observed significant expression of TRPC3 in human smooth muscle from coronary arteries as well as from aorta, we tested the efficiency of a recently discovered TRPC3-selective Ca^2+^ entry blocker, Pyr3 (10 µM) to prevent vascular smooth muscle proliferation and stent implantation-induced hyperplasia of human aorta.

## Methods and results

The effect of Pyr3 on proliferation was measured by detection of BrDU incorporation and PCNA expression in human coronary smooth muscle and microvascular endothelium, which displays significantly smaller expression levels of TRPC as compared to smooth muscle. Pyr3 inhibited smooth muscle proliferation with an IC_50_ of about 3 µM but lacked detectable effects on endothelial proliferation. Measurements of ATP-induced Ca^2+^ signals revealed that Pyr3 suppressed agonist-induced Ca^2+^ entry more effectively in vascular smooth muscle as compared to endothelial cells. Inhibitory effects of Pyr3 on stent implantation-induced arterial injury were tested using a novel *in vitro* model of in-stent hyperplasia in human arteries based on organ-typical culture of human aortic constructs. Pyr3 (10 µM) effectively prevented increases in tissue levels of proliferation markers (PCNA and Ki67) at 2 weeks after stent implantation into human aortae. Similarly, proliferation markers were significantly suppressed when implanting a Pyr3-releasing stent prototype as compared to a bare metal stent control.

## Conclusions

Our results suggest TRPC3 as a potential target for pharmacological control of smooth muscle proliferation. Selective inhibition of TRPC Ca^2+^ entry channels in vascular smooth muscle is suggested as a promising strategy for in-stent restenosis prevention.

